# Sustaining Ecological Functional Zones: The Stabilizing Role of Common Fungi Against Warming Revealed by Altitudinal Transect

**DOI:** 10.3390/jof12030227

**Published:** 2026-03-20

**Authors:** Litao Lin, Guixiang Li, Keming Ma

**Affiliations:** 1 State Key Laboratory of Environmental Criteria and Risk Assessment, Chinese Research Academy of Environmental Sciences, Beijing 100012, China; lin.litao@craes.org.cn; 2 Weifang Academy of Agricultural Sciences, Weifang 261071, China; guixiangli2010@163.com; 3 State Key Laboratory of Urban and Regional Ecology, Research Center for Eco-Environmental Sciences, Chinese Academy of Sciences, Beijing 100085, China

**Keywords:** community stability, elevation, common species, rare species, diversity, network analysis, temperate forest

## Abstract

Fungal communities, typically K-strategy, demonstrate significant potential to counteract environmental stresses. Theories of complexity- and biodiversity-stability suggest that ecosystem stability may be differentially influenced by common species, which engage in intense interactions, and rare species, which contribute to diversity. Here, taking advantage of −0.6 °C/100 m lapse rate, an altitudinal gradient in the Yan-Taihang Mountain Ecological Conservation Area was established, aiming to investigate the responses of common and rare fungi to climatic, plant, and edaphic variations and their potential roles in maintaining stability among low, mid, and high altitudes. Results showed that community composition, rather than diversity, was significantly influenced by altitude, with the abundance of symbiotrophs peaking at mid-altitudes and Saprotrophs at high altitudes. Rare fungi were less accounted for by environmental variables in terms of community composition, whereas their diversity was more sensitive to pH, total phosphorus, and electrical conductivity than the common fungi, indicating that rare species may serve as a resilient gene reservoir under environmental perturbations. The stability of fungal community was further enhanced through interactions among common fungi, with these interactions being slightly compartmentalized and tending more negative at mid (modularity = 0.73, negative-to-positive associations = 0.69%) and high altitudes (modularity = 0.77, negative-to-positive associations = 0.61%) compared with low altitudes (modularity = 0.67, negative-to-positive associations = 0.13%). These results highlighted distinct assembly strategies between common and rare fungi and underscored the importance of common fungi for the persistence of ecological functional zones amidst climate change.

## 1. Introduction

Global environmental changes (e.g., climate warming, soil degradation) pose ongoing threats to the persistence of ecological functional zones through influencing resource availability and acting as an environmental filter [1,2]. Wherein, as a principle component of soil biota, fungal communities maintain close interactions with plants via mycorrhizal associations, pathogenic relationships, and nutrient cycling [3,4,5] and exhibit high resilience to stress [6], thus playing pivotal roles in maintaining ecosystem multifunctionality and stability in the context of environmental changes. Altitudinal gradients, characterized by a lapse rate of −0.6 °C per 100 m and variations in plants and edaphic properties, provide a valuable framework for evaluating the impacts of environmental changes on biotic communities [7]. With increasing altitude, current studies reveal diverse and sometimes contradictory patterns, including increasing trends [8,9], hollow patterns [10], declining trends [11], and no apparent patterns [12,13]. Furthermore, many existing studies have predominantly focused on specific fungal guilds, such as ectomycorrhizal fungi [14,15], arbuscular mycorrhizal fungi [16,17], and saprophytic fungi [18], while the broader variations within soil fungal communities remain underexplored. Understanding mechanisms that regulate soil fungal communities and their interactions along altitudinal gradients is crucial for maintaining ecosystem stability in the face of environmental changes.

The assemblage of fungal communities along altitudinal gradients may be shaped by niche-based deterministic processes [19], such as the filtering effects of climate, vegetation, and edaphic conditions. A global comprehensive study encompassing 235 sites found that climatic factors induce a rapid northward shift in fungal functional groups, with warmer conditions diminishing dominance of ectomycorrhizal fungi in boreal forests, thereby exerting a significant impact on nutrient cycling [20]. Numerous studies have also underscored the substantial influence of vegetation types [21] and tree identity on soil fungal composition [22,23]. Among plant taxa, variations in carbon inputs and root traits (e.g., root morphology and exudation) can distinctly influence the cultivation of fungal communities [24]. Research conducted in alpine meadows demonstrates that specific plant taxa, including *Kobresia* and *Potentilla*, support distinct assemblages of symbiotic and endophytic fungi and serve as indicators of root-associated fungal communities [25]. Edaphic factors, including pH, nutrient availability, and organic matter content, play critical roles in determining fungal fitness and competitiveness, as they profoundly affect enzyme activity and nutrient solubility [26,27,28]. However, within *Quercus*-dominated broadleaf forests, the mechanisms governing the patterns of fungal assembly along altitudinal gradients remain largely unexplored.

Fungal communities typically comprise a few common species with high abundance and numerous rare species with low abundance [29], which may disproportionately influence diversity [30] and interspecific interactions [31]. Specifically, communities with high diversity exhibit complementary and redundant traits and functions and thereby sustaining ecosystem persistence under environmental permutations [32]. Furthermore, network analysis is also a promising avenue for understanding how stress affects interspecific associations (Table 1). Communities with certain interaction patterns (e.g., high modularity and negative/positive associations) could localize the impacts of species extinctions within an interaction module [33] and reduce overlapping responses to environmental permutations [34]. Rare fungi, rather than common ones, are less adapted to local conditions and play a predominant role in shaping the fungal community’s response to climatic, plant, and edaphic variations along the altitudinal gradient [35]. Studies conducted in forests have demonstrated that rare microbial taxa exhibit greater variability in response to environmental changes compared to the common taxa [36,37]. Common taxa may compromise certain functions to enhance their survival and resistance to stressors [35], adopting overlapping strategies and exhibiting a high degree of positive interactions [38]. Communities with high diversity [32] and negative interactions [34] can maintain resilience to environmental changes, such as climate warming, through portfolio and asynchrony effects [30]. Therefore, it is crucial to elucidate the responses of both common and rare fungi to altitudinal gradients in terms of diversity and interactions, in order to enhance our knowledge of the mechanisms underpinning the stability of soil biota in the context of environmental changes.

**Figure 1:**
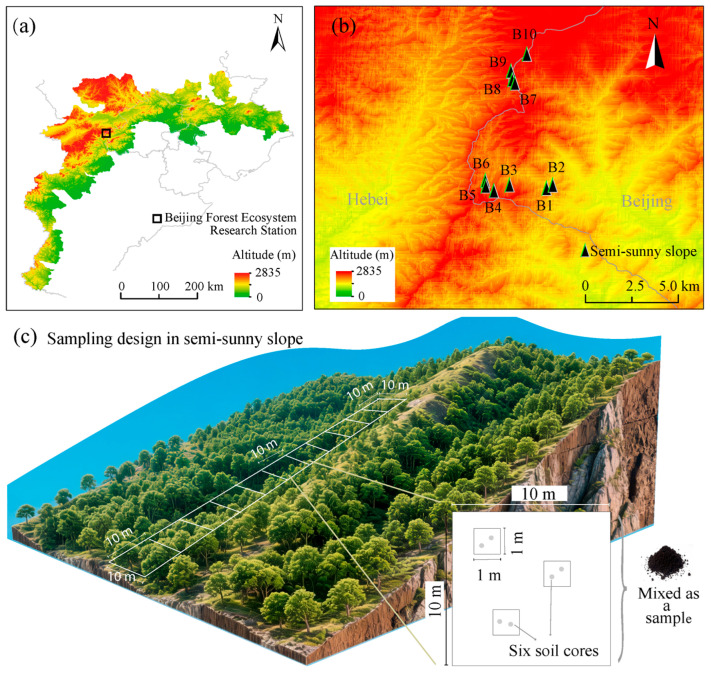
Location of the Beijing Forest Ecosystem Research Station in the Yan-Taihang Mountain Ecological Conservation Area (**a**); the altitudinal gradient composed by ten semi-sunny slopes (**b**); and the sampling design of soil fungi in the semi-sunny slopes (**c**). B1 = 1020–1055 m; B2 = 1065–1160 m; B3 = 1170–1240 m, B4 = 1250–1320 m; B5 = 1330–1395 m; B6 = 1400–1480 m; B7 = 1490–1540 m; B8 = 1546–1600 m; B9 = 1610–1670 m; B10 = 1676–1770 m.

To address the knowledge gap, we selected an altitudinal gradient of oak-dominant forests (1020–1770 m) as the study system, which represents the main forest type of the Yan-Taihang Mountain Ecological Conservation Area [41] (Figure 1). Within the altitudinal range, we investigated fungal communities to evaluate the dominance of common and rare taxa in community assembly and their respective roles in maintaining stability. Given the high persistence of common fungi [37], we posited the following hypotheses: (1) fungal community assembly is more significantly influenced by edaphic and plant conditions than by climatic conditions in terms of species richness and community composition; (2) rare fungi rather than common fungi exhibited greater sensitivity to environmental changes along the altitudinal gradient; and (3) common and rare fungi employ distinct stability strategies, characterized by interspecies associations and diversity, respectively.

## 2. Materials and Methods

### 2.1. Study Area and Design

The study site was located at the Beijing Forest Ecosystem Research Station, affiliated with the Chinese Academy of Sciences (30°57′29″ N, 115°25′33″ E), within the central region of the Yan-Taihang Ecological Conservation Area (Figure 1a,b). This area is characterized by a warm-temperate monsoon climate, with a mean annual temperature ranging from 5 °C to 11 °C, mean annual precipitation between 500 mm and 650 mm, and a mean annual frost-free period of 195 days. The vegetation in the area comprises 80-year-old secondary forests, consisting of *Quercus wutaishanica*, *Betula* spp., *Acer mono*., etc. An altitudinal gradient, primarily dominated by *Q. wutaishanica*, was established on the western slope of the mountains, covering elevations from 1020 m to 1770 m and encompassing 119 plots (Figure 1c), each with dimensions of 10 m × 10 m. Utilizing multivariate wavelet analysis, these 119 plots were categorized into altitudinal zones (i.e., low altitude, mid altitude, and high altitude) for the purpose of constructing fungal networks [42].

### 2.2. Soil Sampling and Analyses

In August 2013, a detailed survey was conducted involving three herbaceous quadrats (1 m × 1 m) within each 10 m × 10 m plot. Concurrently, six soil cores (3.5 cm in diameter and 10 cm in depth) were collected and combined to form a composite sample (Figure 1c). A subset of this homogenized sample was stored at −80 °C for DNA extraction, while the remaining samples were air-dried for physical and chemical analyses. The soil temperature (ST) and moisture (SM) were measured using an iButton device (1922L, Maxim Integrated, San Jose, CA, USA). Soil pH and electrical conductivity were assessed by analyzing a 2.5:1 water-to-soil ratio. Soil organic carbon and total nitrogen contents were quantified using a C/N analyzer (Vario EL III, Elementar AG Germany, Frankfurt, Germany), while soil total phosphorus was determined through the Mo-Sb colorimetric method [43]. The ratios of soil C:N and C:P were calculated based on the measured values of soil organic carbon, total nitrogen, and total phosphorus. Soil particle size distribution into sand, silt, and clay (U.S. Department of Agriculture) was performed using a Mastersizer 2000 Laser Diffraction Particle Analyzer (Malvern Instruments Ltd., Malvern, UK).

### 2.3. Molecular and Bioinformatics Analyses

Total soil DNA was extracted from 0.25 g of each freeze-dried sample utilizing the MOBIO PowerSoil DNA extraction kit (MO Bio Laboratories, Carlsbad, CA, USA). The quality and concentration of the extracted DNA were assessed using NanoDrop spectrophotometer (Thermo Fisher Scientific, Waltham, MA, USA). The fungal internal transcribed spacer 2 (ITS2) regions of the nuclear ribosomal RNA genes were amplified via polymerase chain reaction (PCR) using the forward primer ITS3 (5′-GCATCGATGAAGAACGCAGC-3′) and reverse primer ITS4 (5′-TCCTCCGCTTATTGATATGC-3′) [44]. The PCR was performed in at a total reaction volume of 25 μL, comprising 4 μL of 5× buffer, 2 U of *Taq* DNA polymerase, 2 μL dNTPs (2.5 mmol·L^−1^), 0.5 μL of each primer (5 mmol·L^−1^), 10 ng template DNA, and dd H_2_O to a final reaction volume of 25 μL. The thermal cycling conditions were as follows: 95 °C for 2 min; 30 cycles at 95 °C for 30 s, 55 °C for 30 s, and 72 °C for 45 s; and a final step at 72 °C for 10 min. The PCR products were analyzed by electrophoresis on 1% agarose gels stained with ethidium bromide and visualized under ultraviolet light. To reduce heterogeneity, three replicates of the PCR products from each sample were pooled. The pooled PCR products were purified using the AxyPrepDNA Gel Extraction Kit (AXYGEN, Union City, CA, USA) and subsequently sequenced on the Illumina PE300 platform (Illumina, San Diego, CA, USA).

The sequence reads were processed with a QIIME toolkit [45] and FLASH [46]. The raw sequence reads were merged using FLASH and quality-filtered in QIIME [45] based on the following criteria: the minimum sequence length ≥ 200 bp (excluding barcode and primer sequences); ambiguous bases ≤ 0; homopolymer length ≤ 10 bp; maximum number of primer or barcode mismatches ≤ 0; and minimum mean quality score ≥ 30 in a window of 50 nt. After quality filtering, the sequences underwent *de novo* chimera detection and were clustered into operational taxonomic units (OTUs) at a 97% shared sequence identity threshold using the USEARCH algorithm. The most abundant sequences within each cluster were selected as the representative sequences. Taxonomic assignments for the OTU sequences were performed using the UC classifier method against the UNITE database. All non-fungal clusters and clusters with <5 reads were excluded from further analysis [47] in order to mitigate the impact of OTUs arising from sequencing errors.

### 2.4. Statistical Analyses

To classify common and rare fungal taxa, a species categorization method based on abundance–frequency data was utilized, employing the Fuzzyq package [29]. The diversity of these taxa was characterized by species richness and calculated using the vegan package [48]. Changes in fungal diversity and community composition to increasing altitude were modeled using general linear models in the nlme package [49], with spatial autocorrelation excluded using the corExp correlation. The effects of altitude and other environmental variables (e.g., soil temperature, soil moisture, pH, TN, TP, herb richness, etc.) on fungal diversity and community composition were determined through multiple regressions in the nlme package [49] and distance-based redundancy analysis (db-RDA) in the vegan package [48], respectively. Model selection was conducted based on variance inflation factor (VIF) and Akaike’s information criterion (AIC) [50] to identify the full model and the optimal models.

To quantify changes in community stability to increasing altitude, three co-occupation networks [39] and three co-occurrence networks [51] were constructed. In co-occupation networks, nodes were common and rare fungal OTUs and edges were determined by species pairs with significantly higher togetherness (i.e., negative interaction) or lower togetherness (i.e., negative interaction) than 999 r2dtable null permutations [52]. Togetherness calculates the degree of species pairs that are co-present or co-absent $\left[\begin{array}{cc} 1 & 0\\ 1 & 0 \end{array}\right]$ across the spatial niches [53]. In co-occurrence networks, nodes were the OTUs that occur ≥ 50% samples and edges denoted Spearman’s correlation coefficients among OTUs with certain thresholds (i.e., |Spearman’s rho| ≥ 0.65 and adjusted *p* ≤ 0.01) in terms of relative abundance [51]. And, co-occurrence networks were visualized using graph_from_adjacency_matrix command in igraph package [54]. Thus, edges in networks (i.e., positive and negative interactions) denoted species associations deviating from random conditions rather than real observed interactions, such as predation, pollination, resource competition, and symbiosis. In alignment with biodiversity- and complexity-stability theories (Table 1), this study referred to diversity and stability-related network topologies, including modularity [1], negative/positive association [55], connectance [40], and niche width [56] to evaluate the community stability in response to disturbances. Specifically, high modularity could localize the impact of species population fluctuations in response to environmental stress on the whole community within interaction modules [1], thus promoting community stability. High negative/positive associations may restrict the cascading impacts of species extinction via reducing symmetric responses of species taxa to environmental permutations [55]. Connectance was the ratio of edges to potential edges among taxa [57], thus characterizing the density and complexity of interspecific associations. The formulas of these indexes were as follows.

$$M=\frac{1}{2m}\sum_{i,j}\left(A_{ij}-\frac{k_{i}{\times k}_{j}}{2m}\right)\delta \left(c_{i},c_{j}\right)$$

where *M* is modularity; *m* is the number of interactions in the network; *A*_ij_ is 1 if species *i* interacts with *j* and 0 otherwise; *k*_i_ and *k*_j_ are the number of interactions of species *i* and *j*, respectively; *k*_i_ × *k*_j_/2*m* is the expected number of interactions between species *i* and *j*; δ(*c*_i_, *c*_j_) is 1 if species *i* and *j* are in the same module and 0 otherwise. The (*A*_ij_ − *k*_i_ × *k*_j_/2*m*) is the probability that species *i* interacts with *j* (i.e., interactions within the same module).

$$Connectance=\frac{Num\;of\;edges}{Num\;of\;potential\;edges}$$

$$Neg:positive\;associations=\frac{Negative\;edges}{Positive\;edges}\times 100\%$$

$$NW=1/\sum {\left(p_{ij}\right)}^{2}$$

where *NW* is ecological niche width; *r* is the sum of niche resources; *p*_ij_ is the ratio of the population of species *i* under niche resource *j* to the total population of species *i*.

## 3. Results

### 3.1. Altitudinal Variation in Soil and Plant Properties

**Figure 2:**
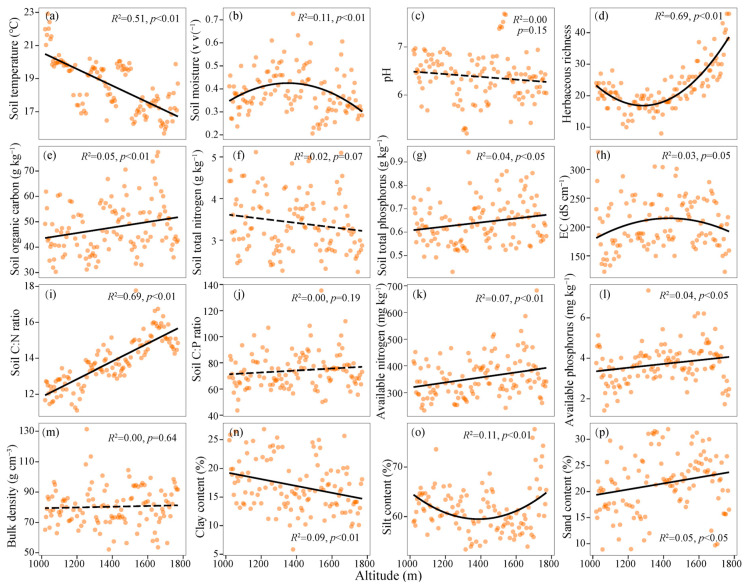
Variation in soil physicochemical properties along the altitudinal gradient. (**a**), soil temperature; (**b**), soil moisture; (**c**), pH; (**d**), herbaceous richness; (**e**), soil organic carbon; (**f**), soil total nitrogen; (**g**), soil total phosphorus; (**h**), electrical conductivity; (**i**), soil C:N ratio; (**j**), soil C:P ratio; (**k**), soil available nitrogen; (**l**), soil available phosphorus; (**m**), soil bulk density; (**n**), soil clay content; (**o**), soil silt content; (**p**), soil sand content. Solid and dashed lines denote significant and non-significant regressions, respectively.

The *Q. wutaishanica*-dominated forests in the Dongling Mountain are mainly distributed between 1020 m and 1770 m (a.l.s.). The altitudinal gradient exhibited a mean soil temperature (ST) of 18.59 °C, soil moisture (SM) of 0.39 v v^−1^, and pH of 6.38, ranging from 22.92 to 15.68 °C, from 0.21 to 0.73 v v^−1^, and from 5.20 to 7.69, respectively (Figure 2a–c). The soil organic carbon (SOC), total nitrogen (TN), and total phosphorus (TP) in these forests were 45.81 [30.28, 77.43] g kg^−1^, 3.42 [2.24, 5.12] g kg^−1^, 0.64 [0.43, 0.94] g kg^−1^, respectively (Figure 2e–g). The soil C:N, C:P, available nitrogen (AN), and available phosphorus (AP) were recorded at 13.81 [11.10, 17.77], 74.15 [43.63, 135.44], 356.7 [233.5, 682.5] mg kg^−1^, and 3.71 [1.43, 7.35] mg kg^−1^, respectively (Figure 2i–l). The electrical conductivity (EC), bulk density (BD), clay content, and herbaceous richness of *Q. wutaishanica* dominated forests were 204.8 [122.3, 330.0] dS cm^−1^, 80.36 [52.18, 131.38] g m^−3^, 16.60 [5.84, 26.83] %, and 22.66 [8.00, 46.00], respectively (Figure 2d,h,m,n).

Along the altitude, ST, SM, SOC, TP, C:N, C:P, EC, AN, AP, soil texture, and herbaceous richness demonstrated significant altitudinal patterns (*p* < 0.05), whereas pH, TN, C:P, and BD did not exhibit significant variation along the altitude (*p* > 0.05) (Figure 2). Notably, ST (*R*^2^ = 0.51, *p* < 0.01) and soil clay content (*R*^2^ = 0.09, *p* < 0.01) were monotonically decreased with increasing altitude (Figure 2a,n). Conversely, SM (*R*^2^ = 0.11, *p* < 0.01) and EC (*R*^2^ = 0.03, *p* = 0.05) displayed hump-back patterns with increasing altitude (Figure 2b,h). SOC (*R*^2^ = 0.05, *p* < 0.01), TP (*R*^2^ = 0.04, *p* < 0.05), AP (*R*^2^ = 0.04, *p* < 0.05), C:N ratio (*R*^2^ = 0.69, *p* < 0.01), and sand content (*R*^2^ = 0.05, *p* < 0.05) exhibited linear increases with rising altitude (Figure 2e,g,i,k,p).

### 3.2. Variation in Fungal Alpha Diversity Along Altitudes

**Figure 3:**
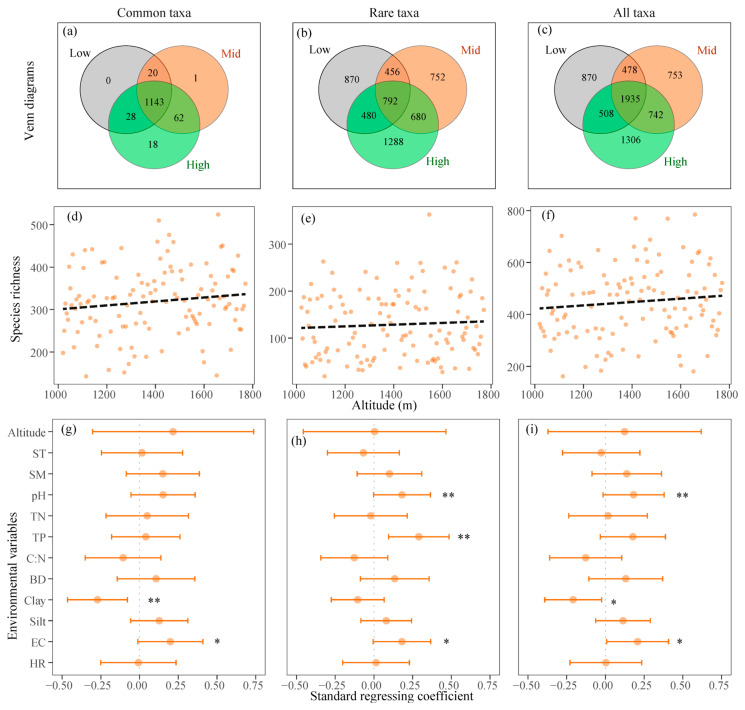
Venn diagrams of fungal taxa observed at low, mid, and high altitudes (**a**–**c**), species richness along the altitudinal gradient (**d**–**f**), and the regression coefficient of species richness against environmental variables (**g**–**i**). (**a**,**d**,**g**), common taxa; (**b**,**e**,**h**), rare taxa; (**c**,**f**,**i**), all taxa. Dashed lines denote insignificant regressions. *, *p* < 0.05; **, *p* < 0.01.

**Figure A1:**
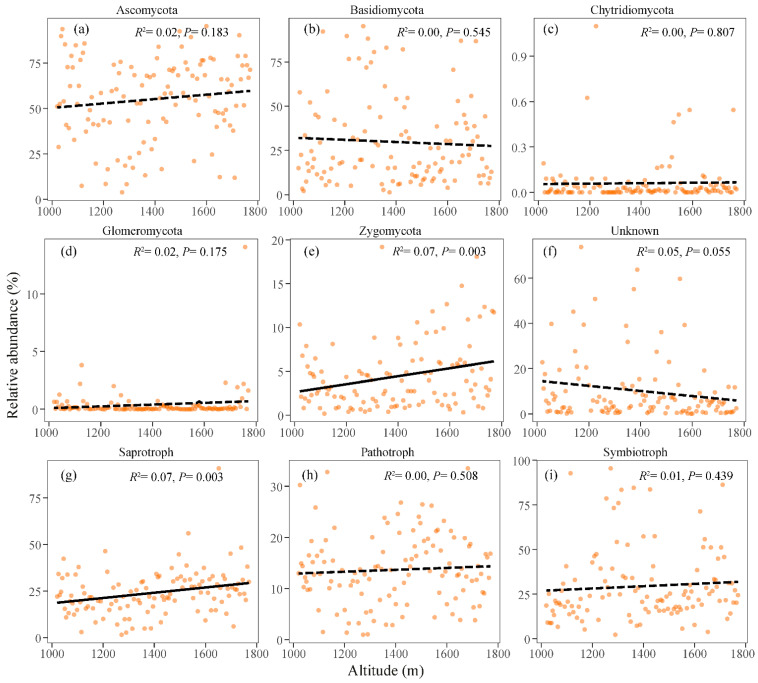
Variation in the relative abundance of fungal phyla along the altitudinal gradient. Solid and dashed lines denote the significant and non-significant regressions, respectively.

**Figure A2:**
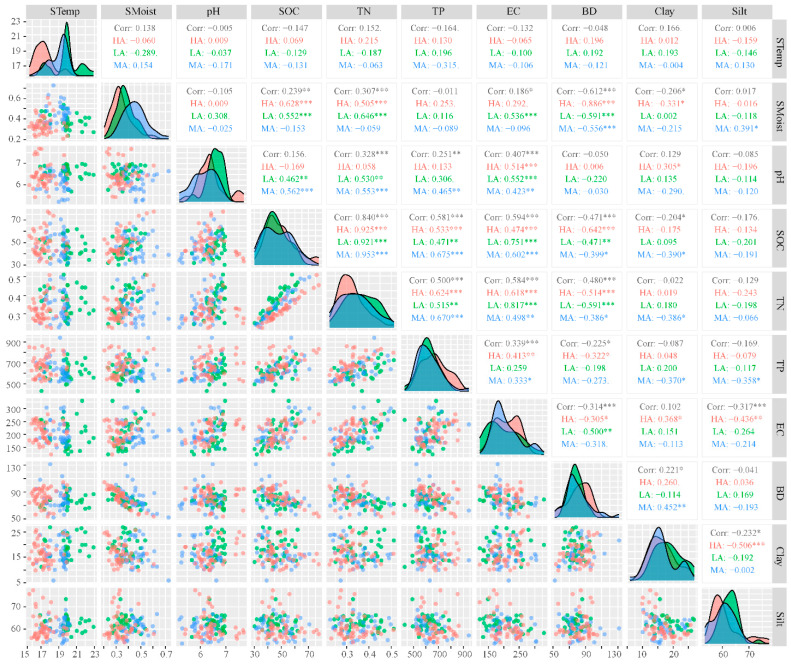
Pearson’s relationships between soil physicochemical properties. STemp, soil temperature; SMoist, soil moisture; SOC, soil organic carbon; TN, soil total nitrogen; TP, soil total phosphorus; EC, electrical conductivity. LA, low altitude; MA, mid altitude; HA, high altitude. *, *p* < 0.05; **, *p* < 0.01; ***, *p* < 0.001.

Across 119 samples, 7368 fungal OTUs were identified, comprising 6094 OTUs classified as rare taxa and 1274 as common taxa, respectively (Figure 3a–c). Across varying altitudes, the species richness of common and rare taxa exhibited a non-significant variation pattern with increasing altitude (*p* > 0.05) (Figure 3d–f) and non-significant correlations with increasing soil temperature (Figure 3g–i, Table A1). The species richness of all taxa was significantly promoted with increasing pH and EC and reducing soil clay content (Figure 3i, Table A1). Specifically, the species richness of common taxa, as well as all taxa, was significantly promoted with reducing soil clay content (*p* < 0.01) (Figure 3g–i, Table A1). In contrast, the species richness of rare fungi was significantly promoted with increasing pH (*p* < 0.01) and TP (*p* < 0.01) and demonstrated a steeper relationship with EC (*r* = 0.281, *p* < 0.01) compared to the common (*r* = 0.218, *p* < 0.05) (Figure 3g–i, Table A1). The SOC and TN, which significantly correlated with TP and EC (*r* > 0.500, *p* < 0.001) (Figure A2), also demonstrated a steeper relationship with the species richness of rare fungi (*r* = 0.230 and *r* = 0.259) compared to the common fungi (*r* = 0.196 and *r* = 0.197) (Figure A1).

### 3.3. Changes in Community Composition Along Altitudes

**Figure 4:**
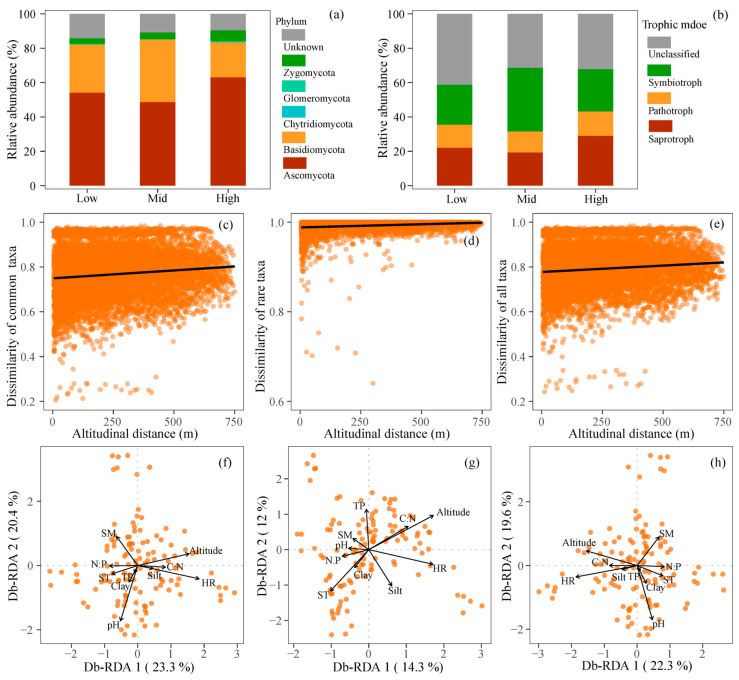
Relative abundance of fungal phyla and functional groups (**a**,**b**), responses of fungal community composition to altitudinal difference (**c**–**e**), distance-based redundancy analysis of community composition against environmental factors (**f**–**h**). (**a**), fungal phyla; (**b**) fungal functional groups; (**c**,**f**), common taxa; (**d**,**g**), rare taxa; (**e**,**h**), all taxa. Solid lines in (**c**–**e**) denote significant regressions. ST, soil temperature; SM, soil moisture; SOC, soil organic carbon; TN, soil total nitrogen; TP, soil total phosphorus; EC, electrical conductivity; HR, herbaceous richness.

The fungal communities were predominantly composed of phyla Ascomycota, Basidiomycota, Zygomycota, and Glomeromycota, exhibiting relative abundances of 56.95%, 26.60%, 4.87%, and 0.44%, respectively (Figure 4a, Table 2). The relative abundance of saprotrophic, pathotrophic, and symbiotrophic fungi were 24.70%, 13.47%, and 27.62%, respectively (Figure 4b, Table 2). The relative abundances of Ascomycota, Basidiomycota, Zygomycota, Saprotroph, and symbiotroph significantly differed across LA, MA, and HA (Figure 4a,b, Table 2). Specifically, the relative abundances of Ascomycota (63.09%), Zygomycota (6.27%), and Saprotrophs (29.10%) were significantly elevated at HA compared to those at LA and MA (48.68–54.18%, 3.24–3.89%, and 19.32–22.09%, respectively) (*p* < 0.05) (Figure 4a,b, Table 2). In contrast, the relative abundances of Basidiomycota (36.38%) and symbiotrophs (37.08%) peaked at MA (*p* < 0.05), surpassing those observed at LA and HA (20.36–27.95% and 23.23–24.55%) (Figure 4a,b, Table 2).

For phylum Ascomycota, the relative abundances of genera *Gibberella* (3.660%), *Phoma* (2.413%), *Preussia* (1.413%), *Ilyonectria* (1.227%), *Fusarium* (0.504%), *Archaeorhizomyces* (0.422%), *Nectria* (0.315%), and *Paraphoma* (0.989%) were significantly highest at HA, whereas genera *Capronia* (5.140%), *Exophiala* (3.737%), *Humicola* (3.596%), *Cladophialophora* (2.525%), and *Pseudogymnoascus* (0.549%) peaked at MA (*p* < 0.05) (Table A2). For phylum Basidiomycota, the relative abundances of genera *Cortinarius* (18.288%) and *Tomentella* (1.271%) peaked at MA (*p* < 0.05), whereas genera *Cryptococcus* (2.637%), *Boletus* (2.530%), *Geminibasidium* (2.174%), and *Scleroderma* (0.734%) exhibited the highest relative abundances at HA (Table A2). Ectomycorrhizal fungi exhibited a significantly higher relative abundance at MA (22.940%) compared to those at LA (8.418%) and HA (6.818) (Table A3). The relative abundance of wood and plant Saprotrophs (2.157%) and plant Saprotrophs (6.174%) were significantly higher at HA than those at LA (1.784% and 3.172%) and MA (1.097% and 3.804%), respectively (Table A3).

For fungal OTUs, the RDA result showed that the community composition of common fungi was more accounted by the first two axes (53.7%) compared to the rare fungi (26.3%). Alternations in community composition showed a significantly linear relationship with altitudinal distance (*p* < 0.05), with a steeper slope detected in common taxa (*β*-weight = 0.011) compared to rare taxa (*β*-weight = 0.053) (Figure 4c–e). The community composition of soil fungi, as well as the common and rare fungi, was significantly influenced by SM, pH, TN, TP, and herb richness (*p* < 0.05) (Figure 4f–h, Table A4). Furthermore, the composition of common taxa was also significantly affected by soil clay and EC, whereas that of rare taxa was significantly influenced by ST and silt content (Figure 4f–h, Table A4).

### 3.4. Changes in Interspecific Interaction Along Altitudes

**Figure 5:**
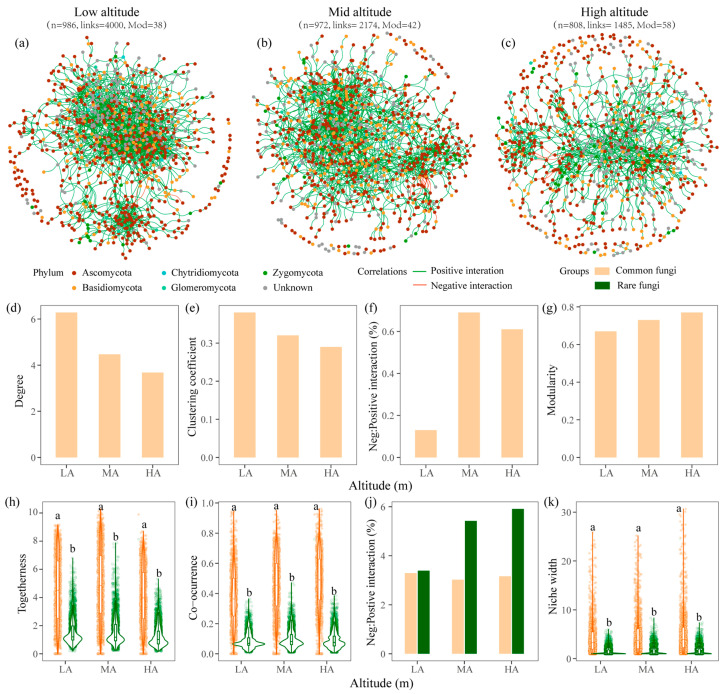
Visualization (**a**–**c**) and topologies of fungal co-occurrence networks (**d**–**g**), and the topologies of fungal co-occupation networks along the altitudinal gradient (**h**–**k**). LA, low altitudes; MA, mid altitudes; HA, high altitudes. Different lowercase letters in (**h**–**k**) denote 0.05 singificant ANOVA differences between common and rare fungi.

From the perspective of co-occurrence, three networks were constructed for low (LA), mid (MA), and high altitudes (HA), respectively (Figure 5a–c). The topologies of co-occurrence networks across varying altitudes revealed enhanced community stability at MA and HA compared to LA (Figure 5a–c). In particular, the fungal network at LA demonstrated a lower negative/positive interactions (NPI = 0.13%) and a higher clustering coefficient (CC = 0.38) compared to the MA (NPI = 0.69%, CC = 0.32) and HA (NPI = 0.61%, CC = 0.29) (Figure 5e, Table A5). The network modularity at low altitude (0.67) was marginally lower than that observed at the MA (0.73) and HA (0.77), suggesting a more modular structure at HA (Figure 5d–g, Table A5). The increased modularity and moderate proportion of negative interactions at MA (0.69%) and HA (0.61%) indicate more stable interaction patterns, thereby improving adaptation to environmental perturbations.

From the perspective of co-occupation, networks demonstrated that common fungi predominantly influenced interspecific interactions, with both the degree and niche width of common fungi being significantly greater than those of rare fungi (*p* < 0.05) (Figure 5i–k, Table 3). Specifically, the connectance of common taxa at LA, MA, and HA was 17.14%, 20.09%, and 19.13%, respectively, whereas for rare fungi, it was only 4.33%, 4.89%, and 4.59%, respectively (Figure 5i–k, Table 3). Moreover, common fungi exhibited considerable higher niche width (4.42–5.24) compared to the rare (1.42–1.59). Along the altitudinal gradient, the negative/positive interactions among common and rare fungal species increased markedly at MA (5.55) and HA (5.95) compared to LA (4.96) (Figure 5k, Table 3). These findings suggest that common fungi may play a dominant role in shaping interactions of fungal communities.

## 4. Discussion

Fungal communities are composed of both common (*n* = 1274) and rare species (*n* = 6094), each exhibiting distinct strategies in response to environmental changes and playing different roles in community stability. Our study found that community composition, rather than diversity, was significantly influenced by altitude (*p* < 0.05), with the relative abundance of Ascomycota, Zygomycota, and Saprotrophs highest at HA (*p* < 0.05) and the relative abundance of Basidiomycota and symbiotrophs highest at MA (*p* < 0.05). By distinguishing common and rare species based on their abundance and frequency, we demonstrated that rare species predominantly drove fungal turnover across LA, MA, and HA and were more sensitive to environmental factors in terms of diversity. In contrast, common species were more influenced by environmental factors in terms of community composition. Common species contributed to the stability of fungal communities at HA through compartmentalized interactions, with diversity and modularity slightly increasing and the clustering coefficient decreasing as altitude increased. These results enhance our knowledge of distinct strategies between common and rare species during community assembly and highlight the crucial role of common species in ensuring community stability.

### 4.1. Fungal Community Composition Not Diversity Significantly Altered with Altitude

The diversity of fungal communities exhibited an upward trend with increasing altitude (*r* = 0.106, *p* = 0.251) and was significantly influenced by pH (*β*-weight = 0.18), EC (*β*-weight = 0.21), and soil texture (*β*-weight = 0.20), rather than by climatic and plant properties (Figure 3). Compared with the typically R-strategy bacteria exhibiting a decreasing pattern [58], our results suggested that variations in microbial diversity with increasing altitude could be heterogeneous due to the diverse ecologies of microbial taxa and not constrained by climatic factors. This altitudinal result contrasts with the decreased patterns observed in bacteria [59] or the hump-shaped patterns of invertebrates along this altitudinal gradient [58]. However, it aligns with findings by Peay et al. [8] and Ni et al. [9], demonstrating that fungal diversity increases monotonically with altitude. On one side, critical factors, such as pH and EC, imposed physiological constraints on abundant fungal hyphae and exhibited inconsistent variation as altitude increased (Figure 2), thereby diminishing the influence of altitude on fungal diversity. Previous studies have indicated that acidification (i.e., high H^+^ ion) hindered the processes of NH_4_^+^, K^+^, and Ca^2+^ exchanges across cell membranes [60]. Additionally, low EC can reduce the absorption of nitrogen, phosphorus, and dissolved organic carbon nutrients due to a slower rate of soil substance migration [61]. An increase in pH and EC could alleviate the leaching of Ca^2+^/Mg^2+^ ions and stabilize soil organic matter and fertility (Figure A2), thereby enhancing the availability of substances necessary for fungal growth [62]. Similarly, Liu et al. [63] suggested that soil pH positively influences fungal diversity in Southwestern China, with no significant trends observed with increasing altitude. On the other side, clay content directly influences soil physical structure (i.e., soil porosity), thereby affecting the spatial niche availability for fungal hyphae. The common fungi that have relatively high population sizes and hyphae lengths, rather than rare fungi, were negatively correlated with clay content regarding species richness. Fungal hyphae navigate the porous spaces within soil [64], and an elevated sand content can enhance soil porosity, thereby facilitating the development of fungal hyphae and plant roots.

Fungal composition, rather than species richness, was significantly influenced by altitude (Figure 4), with Ascomycota, Zygomycota, and Saprotrophs being most abundant at HA (*p* < 0.05) and Basidiomycota and symbiotrophs highest at MA in terms of relative abundance (*p* < 0.05) (Table 2). The increases in Ascomycota and Saprotrophs at HA suggest a notable selection for stress tolerance and organic matter mineralization functions within fungal communities. At HA, chilling stress enhanced energy allocation towards defensive traits, such as increased cell wall thickness [65], thereby augmenting the abundance of Ascomycota (Figure 2a). Consistent with our findings, previous studies by Peay et al. [8] and Lin et al. [66] have also highlighted the significant role of Ascomycota in coping with environmental stresses. Furthermore, chilling conditions favored saprotrophic fungi over bacteria due to their high efficiency in organic matter mineralization, thereby safeguarding forest nutrient cycling at HA [25]. Evidence from alpine meadow and Antarctic soil also supports the observation that the relative abundance and species richness of saprotrophic fungi decrease with rising temperatures [25,65]. Conversely, most symbiotrophs were classified as Basidiomycota and their abundance reached its peak at MA (Table 2), favoring symbiotrophs with efficient resource acquisition capabilities. Particularly, the relative abundances of ectomycorrhizal fungi (22.94%) and the genus *Cortinarius* (18.29%) were significantly higher at MA compared to the LA (8.42%, 4.75%) and HA (6.82%, 4.89%) (Table A2 and Table A3). At MA, optimal temperature and moisture conditions promoted symbiotroph-mediated nutrient-carbon exchanges, facilitated by increased plant growth. Consistent with the findings, Gao et al. [67] reported that mycorrhizal fungi in wetter valleys exhibit significantly higher richness values compared to those in ridge habitats. The highest SM and EC at MA (Figure 1) may enhance hyphal exploration and nutrient transfer in symbiotrophs due to increased ion mobility [62]. At MA, optimal C:N ratios ~14.3 (Figure 1) facilitated efficient carbon utilization and nitrogen conservation, thereby achieving a balance between carbon and nutrient resources for hyphal expansion [68]. Furthermore, fungal communities exhibited significant distance–decay relationships in terms of community composition, with fungal dissimilarity being strongly correlated with altitudinal distance (Figure 4b, Table A4). Similarly, Miyamoto et al. [14] found that the beta diversity of fungal communities is significantly influenced by altitude, suggesting that stochastic dispersal processes play a role in the regulation of fungal communities. Meta-analyses across 13 sites indicated that ectomycorrhizal fungal communities were significantly associated with spatial vectors, exhibiting an average community dissimilarity of 0.740~0.993 [69]. From both taxonomic and functional perspectives, these findings supported the niche–neutral continuum hypothesis, positing that fungal communities are structured by a combined effect of neutral processes and ecological niche at the landscape scale.

### 4.2. Rare Fungi Dominate Diversity While Common Fungi Dominate Composition Responses to Environment

This study found a clear functional dichotomy between common and rare fungal taxa in their responses to altitudinal gradients and the associated environmental factors (e.g., climatic, plant, and edaphic factors). Rare fungi demonstrated a greater degree of variation across low, mid, and high altitudes and exhibited a more pronounced response to soil EC, pH, SOC, TN, and TP than the common fungi in terms of species richness (Figure 3b, Table A1). This differential sensitivity supports the second hypothesis that rare fungi rather than common fungi are more responsive to environmental changes, thereby highlighting the unique ecological strategies of the rare biosphere [70]. In contrast to the high adaptability and large population size of common fungi, rare fungi had smaller population abundances and occupied specialized and corner niche habitats [71,72]. Variations in specific chemical conditions, such as pH and phosphorus availability, can create micro-niches that allow subsets of the rare biosphere to flourish, thereby enhancing local species richness without drastically altering the overall community composition dominated by common fungi [73]. The lack of response to clay content further emphasizes that the distribution of rare fungi is less constrained by broad physical habitat filters and more influenced by specific chemical and nutritional gradients [3,70]. This makes the diversity of rare fungi serve as a highly sensitive indicator of environmental change, capturing subtle variations that are not evident in the diversity patterns of more stable common taxa [72]. This functional differentiation is crucial for understanding ecosystem responses, as it implies that monitoring solely community composition may overlook early warning signals of environmental change encoded in the dynamics of the rare biosphere.

Common fungi, as the dominant constituents, demonstrated a greater variation in slope and explanation rate to environment changes in terms of community composition (Figure 4). This finding robustly supports the paradigm that common fungi are the principal architects of community composition, whereas rare fungi are the key responders shaping diversity-environment relationships (Figure 3, Table A1). On one side, common fungi occupied broader niches and exhibited a stronger distance–decay relationship along altitudinal gradients compared to rare fungi (Figure 4c,d), indicating a distinct turnover in their community structure along the altitudinal gradient [69]. On the other hand, the substantial variation in common fungi was further corroborated by the db-RDA results (Table A2), which demonstrated a high explanation rate regarding environmental changes, thereby indicating a strong influence of deterministic processes such as environmental filtering [74]. Specifically, the composition of common taxa is significantly influenced by key abiotic factors, including soil clay content and electrical conductivity (EC). These factors serve as fundamental habitat templates that determine soil structure, water retention, and ionic strength [75]. Common taxa, due to their high biomass and prevalence, likely possess broader ecological niches and exhibit greater competitiveness for dominant resources [71]. As a result, shifts in these overarching environmental conditions directly filter which common species can persist [35], thereby disproportionately driving the compositional trajectory of the entire fungal community. The observed significant changes in the relative abundance of major phyla, such as Zygomycota, along the altitudinal gradient (Table 2) largely reflect the responsive nature of these common taxa.

### 4.3. Distinct Stabilizing Mechanisms of Common and Rare Fungi at Mid and High Altitude

Using network analyses, this study indicated that the increases in stability of fungal communities at MA and HA might emerge from distinct contributions of the common and rare members, with common taxa contributing to robustness and rare taxa enhancing resilience, respectively. Compared with temporal stability of communities in previous studies [76], this study, from the perspective of interaction network structure, revealed critical roles of common fungi in stabilizing community structure. Specifically, the modularity and negative/positive associations among common taxa at MA and HA were relatively higher compared to those at LA (Figure 5), thus localizing the effects of disturbances and reducing the risks of population fluctuations and species extinction on the entire community (Table 1). On one side, common fungi at MA and HA might exhibit relatively long generation cycles due to lower temperatures, which may facilitate niche expansion and niche co-occupation rather than intensifying interactions to maintain community functions [71]. Wang et al. [70] reported that common fungi demonstrated significant resistance in spatially complex environments and are less affected by environmental changes. Wu et al. [40] observed that warming shifted fungal co-occurrence networks towards greater complexity and reduced modularity in permafrost ecosystems. On the other side, common fungi with high abundance were the primary contributors to organic carbon decomposition and nutrient cycling within the community [71], with symbiotrophs (e.g., ectomycorrhizal fungi) and Saprotrophs peaking at MA and HA, respectively (Figure 4, Table 2 and Table A3), thereby stabilizing plant nutrient uptake and litter composition functions. With decreasing SOC content and quality (Figure 2 and Figure 5a, Table A4), common fungi may depend more on positive associations to enhance the function of organic carbon mineralization [55]. Therefore, preserving microbial associations is crucial for mitigating the adverse effects of altitude-induced biodiversity loss on ecosystem functions [1], thereby ensuring community structure and persistence.

The study demonstrated that fungal diversity remained unaffected by increasing altitude, with rare fungi exhibiting greater sensitivity to environmental variables compared to the common fungi (Figure 3 and Figure 4), thereby augmenting community function and the capacity to recover from disturbances [72]. Rare fungi, persisting at low abundance, primarily occupy narrow and specialized niches inaccessible to common taxa (Figure 5). Their persistence at MA and HA optimized resource utilization and enhanced community function [77]. The elevated beta diversity observed among rare fungi indicates a more spatially heterogeneous and stochastic community assembly [78], contributing to an expanded regional species pool. This pool serves as a substantial genetic reservoir of colonists for local habitats following disturbances [78], enabling certain rare taxa to rapidly increase in abundance in response to environmental perturbations (e.g., pH or nutrient pulse) [79]. This is evidenced by their pronounced response to variables such as soil temperature and silt content (Table 2), which are generally more dynamic than the factors influencing common taxa. Consequently, rare fungi likely contribute to ecosystem stability through resilience mechanisms, enhancing the community’s capacity to recover from perturbations, and thereby sustaining ecosystem functions.

Using the altitudinal proxies, temperature, plant, and edaphic factors co-varied with altitudes and exerted a pronounced impact on both common and rare taxa, through which different nutrient inputs and microhabitats provided by changes in plant composition simultaneously regulate the stable core of common fungi and activate different subsets of the rare fungal seed bank. Due to a lapse rate of −0.6 °C per 100 m, the higher network stability at MA and HA than LA (Figure 5) was a finding crucial for predicting ecosystem responses to future global change scenarios. Future studies could elucidate the distinct effects of climate warming, plant, and edaphic factors which co-vary with the altitudes on the stability of microbial communities.

## 5. Conclusions

Utilizing altitudinal proxies, this study indicated that environment-driven shifts alongside increasing altitude were characterized by an increase in Ascomycota, Zygomycota, and Saprotrophs at high altitudes and a predominance of Basidiomycota and symbiotrophs at mid altitudes, thus potentially altering ecosystem processes. Employing an abundance-frequency-based method for classifying common and rare taxa, we identified a distinct sensitivity of these fungal groups to environmental factors as altitude increased. Specifically, common fungi predominantly influenced community composition, whereas rare fungi primarily contributed to community diversity. Common and rare fungi may exhibit a dual mechanism in maintaining community stability, with common taxa enhancing resilience via robust interactive networks and rare taxa supporting adaptive capacity through high diversity and phylogenetic structure. At mid and high altitudes, common fungi foster stability through the relative abundance of functional groups, modularity, and negative-to-positive interactions, which localize the effects of disturbances and support community persistence. At low altitudes, the amplification of interactions among common fungi may play a crucial role in mitigating the adverse effects of warming-induced biodiversity loss on ecosystem functions, albeit potentially at the cost of reduced community stability. Future studies could elucidate roles of climate conditions and the co-varied plant and edaphic properties in stabilizing communities within temperate broadleaf forests. And, the management of ecological functional zones in the context of climate change should account for the distinct stabilizing roles of common and rare fungi within the community.

## Figures and Tables

**Table 1:** Community stability theories, pathways, and the relationship with common and rare species.

Theories	Regulatory Pathways	Roles of Common and Rare Species	Reference
Biodiversity stability	Complementary Effect: Functional traits of different species complement one another, resulting in more efficient and stable utilization of resources	Rare taxa serve as a gene reservoir; functional stability	[28,32]
	Asynchrony Effect: A diverse array of species offers varied responses to environmental fluctuations, akin to the benefits of a diversified investment portfolio.	Rare taxa serve as a gene reservoir; functional stability	[32,39]
	Sampling Effect: An increased number of species enhances the likelihood of incorporating highly productive and stable species.	Common taxa served as keystones, and rare taxa as a gene reservoir; functional stability	[2,32]
Complexity stability	Modularity Effect: Species is organized into relatively independent modules, thereby localizing the effects of disturbances.	Common taxa more engaged in interactions; structural stability	[1,34]
	Interaction Intensity: Numerous negative or weak interactions function as “buffers”, stabilizing fluctuations induced by a limited number of strong interactions, e.g., connectance, negative/positive interaction.	Common taxa more engaged in interactions; structural stability	[34,40]
	Interaction Pattern: Interactions adhere to specific patterns, e.g., cluster coefficient, nestedness.	Common and rare taxa; structural stability	[3,34]

**Table 2:** Mean [−0.95 CI, 0.95 CI] relative abundance of fungal phyla and functional groups. Different lowercase letters denote significant LSD difference α = 0.05 among low, mid, and high altitudes.

Groups		Low Altitude	Mid Altitude	High Altitude
Phylum	Ascomycota	54.18 [47.15, 61.20] b	48.68 [41.93, 55.42] b	63.09 [58.02, 68.17] a
	Basidiomycota	27.95 [20.25,35.65] ab	36.38 [28.98, 43.76] a	20.36 [14.80, 25.92] b
	Chytridiomycota	0.08 [0.03, 0.12] a	0.02 [0, 0.07] a	0.08 [0.05, 0.11] a
	Glomeromycota	0.38 [0, 0.83] a	0.20 [0, 0.63] a	0.60 [0.28, 0.93] a
	Zygomycota	3.24 [1.82, 4.66] b	3.89 [2.53, 5.26] b	6.27 [5.24, 7.30] a
	Unknown phylum	14.17 [9.17, 19.16] a	10.83 [6.04, 15.62] a	9.59 [5.98, 13.20] a
Function	Saprotroph	22.09 [18.50, 25.68] b	19.32 [15.87, 22.77] b	29.10 [26.51, 31.70] a
	Pathotroph	13.42 [11.22, 15.62] a	12.27 [10.16, 14.37] a	14.18 [12.59, 15.77] a
	Symbiotroph	23.23 [16.98, 29.48] b	37.08 [31.08, 43.08] a	24.55 [20.03, 29.07] b
	Unclassified guild	41.26 [35.37, 47.15] a	33.34 [35.37, 47.15] b	32.17 [27.91, 36.43] b

**Table 3:** Topologies of fungal co-occupation networks with increasing altitudes. LA, low altitudes; MA, mid altitudes; HA, high altitudes.

Topologies	Low Altitude			Mid Altitude			High Altitude		
	Both	Common	Rare	Both	Common	Rare	Both	Common	Rare
Connectance	17.14%	18.81%	4.33%	20.09%	22.51%	4.89%	19.43%	24.12%	4.57%
Degree	618.60	224.02	112.61	937.61	275.92	130.94	823.57	301.75	148.05
Positive edges	2,343,894	266,818	292,556	2,902,846	338,285	350,924	3,698,648	377,488	479,680
Negative edges	116,204	8789	11,447	160,986	10,219	19,018	220,626	11,935	28,731
Negative/positive edges (%)	4.96	3.29	3.91	5.55	3.02	5.42	5.97	3.16	5.99
Togetherness	2.49	4.53	1.56	2.66	4.90	1.63	2.05	4.09	1.25
Niche width	2.36	4.42	1.42	2.63	4.93	1.57	2.61	5.24	1.59

## Data Availability

The original contributions presented in this study are included in the article. Further inquiries can be directed to the corresponding author.

## Appendix A

**Table A1:** Pearson’s correlations of fungal diversity against plant and soil properties. SOC, soil organic carbon; TN, soil total nitrogen; TP, soil total phosphorus; EC, electrical conductivity. Values in bold are significant correlations.

Variables	Common Taxa		Rare Taxa		All Taxa	
	r	p	r	p	r	p
Altitude (m)	0.131	0.156	0.060	0.517	0.106	0.251
Soil temperature (°C)	−0.082	0.373	−0.095	0.306	−0.095	0.302
Soil moisture (v v^−1^)	0.140	0.129	0.020	0.832	0.091	0.324
Tree abundance	−0.041	0.654	−0.083	0.367	−0.066	0.476
Tree richness	−0.067	0.472	0.039	0.673	−0.019	0.837
Herb abundance	0.023	0.808	−0.142	0.124	−0.058	0.530
Herb richness	0.029	0.752	−0.002	0.982	0.016	0.864
SOC (g kg^−1^)	**0.196**	**0.032**	**0.230**	**0.012**	**0.230**	**0.012**
TN (g kg^−1^)	**0.197**	**0.031**	**0.259**	**0.004**	**0.245**	**0.007**
TP (g kg^−1^)	0.148	0.108	**0.368**	**0.000**	**0.271**	**0.003**
pH	0.160	0.083	**0.294**	**0.001**	**0.240**	**0.008**
EC (ds cm^−1^)	**0.218**	**0.017**	**0.281**	**0.002**	**0.267**	**0.003**
C:N ratio	0.010	0.911	−0.026	0.782	−0.007	0.941
Bulk density (g cm^−3^)	−0.115	0.213	−0.043	0.642	−0.088	0.339
Clay content (%)	**−0.286**	**0.002**	−0.094	0.311	**−0.213**	**0.020**
Silt content (%)	0.079	0.393	−0.006	0.947	0.043	0.644

**Table A2:** Mean [−0.95 CI, 0.95 CI] relative abundance of fungal genera. Different lowercase letters and the bold font denote significant LSD difference α = 0.05 among low, mid, and high altitudes.

Phylum	Genus	Low Altitude	Mid Altitude	High Altitude
Ascomycota	Alatospora	0.110 [0.000, 0.268]	0.076 [0.000, 0.227]	0.170 [0.056, 0.284]
	**Alternaria**	**0.137 [0.043, 0.232] ab**	**0.051 [−0.039, 0.142] b**	**0.195 [0.126, 0.263] a**
	Apodus	0.067 [0.000,0.134]	0.132 [0.067, 0.197]	0.133 [0.084, 0.182]
	**Archaeorhizomyces**	**0.107 [−0.182, 0.396] ab**	**0.062 [−0.215, 0.339] b**	**0.422 [0.213, 0.631] a**
	Aspergillus	0.042 [−0.206,0.289]	0.070 [−0.168, 0.307]	0.169 [0.000, 0.348]
	Aureobasidium	0.605 [0.113,1.097]	0.064 [0.000, 0.536]	0.185 [0.000, 0.540]
	**Capronia**	**6.283 [5.111, 7.455] ab**	**5.140 [4.015, 6.264] b**	**3.960 [3.114, 4.807] a**
	Cephaliophora	0.003 [0.000,0.532]	0.000 [0.000, 0.000]	0.390 [0.007, 0.772]
	Chalara	0.044 [0.000,0.393]	0.130 [0.000, 0.465]	0.462 [0.209, 0.714]
	**Cladophialophora**	**1.634 [1.092, 2.177] b**	**2.525 [2.005,3.046] a**	**1.279 [0.887, 1.672] b**
	Cladosporium	0.287 [0.005, 0.570]	0.296 [0.025, 0.567]	0.588 [0.384, 0.792]
	Cylindrocarpon	0.215 [0.138, 0.291]	0.170 [0.097, 0.243]	0.166 [0.111, 0.221]
	Dictyochaeta	0.082 [0.000, 0.352]	0.210 [0.000, 0.469]	0.197 [0.002, 0.392]
	**Exophiala**	**2.434 [1.716, 3.151] b**	**3.735 [3.046, 4.423] a**	**2.460 [1.942, 2.978] b**
	**Fusarium**	**0.201 [0.001, 0.401] b**	**0.138 [0.000, 0.330] b**	**0.504 [0.359, 0.648] a**
	**Gibberella**	**1.120 [0.104, 2.137] b**	**1.198 [0.223, 2.174] b**	**3.660 [2.925, 4.395] a**
	Herpotrichia	0.038 [0.000, 0.334]	0.142 [0.000, 0.426]	0.309 [0.095, 0.523]
	**Humicola**	**1.831 [0.965, 2.698] b**	**3.596 [2.765, 4.428] a**	**2.193 [1.566, 2.819] b**
	Hymenoscyphus	0.027 [0.000, 0.326]	0.065 [0.000, 0.353]	0.389 [0.172, 0.605]
	**Ilyonectria**	**0.865 [0.568, 1.163] b**	**0.867 [0.581, 1.152] b**	**1.227 [1.012, 1.442] a**
	Lachnum	0.179 [0.000, 0.513]	0.215 [0.000, 0.536]	0.239 [0.000, 0.480]
	Leohumicola	0.883 [0.473, 1.292]	0.939 [0.546, 1.332]	0.914 [0.618, 1.210]
	**Leptosphaeria**	**0.167 [0.011, 0.323] b**	**0.188 [0.038, 0.338] b**	**0.497 [0.384, 0.61] a**
	Lophiostoma	0.407 [0.000, 0.869]	0.240 [0.000, 0.683]	0.728 [0.394, 1.062]
	**Mycoarthris**	**1.264 [0.671, 1.857] a**	**0.120 [0.000, 0.689] b**	**0.072 [0.000, 0.501] b**
	Mycocentrospora	0.079 [0.000,0.763]	0.037 [0.000, 0.693]	0.644 [0.149, 1.138]
	**Nectria**	**0.031 [0.000, 0.177] ab**	**0.223 [0.083, 0.363] b**	**0.315 [0.209, 0.42] a**
	Neonectria	0.168 [0.091, 0.246]	0.178 [0.104, 0.253]	0.244 [0.188, 0.301]
	**Paraphoma**	**0.268 [0.024, 0.511] b**	**0.264 [0.031, 0.498] b**	**0.989 [0.813, 1.165] a**
	Penicillium	0.598 [0.38, 0.816]	0.419 [0.209, 0.628]	0.423 [0.266, 0.581]
	Phaeosphaeriopsis	0.020 [0.000, 0.408]	0.379 [0.007, 0.752]	0.039 [0.000, 0.319]
	**Phoma**	**0.897 [0.000, 2.071] b**	**0.557 [0.000, 1.684] b**	**2.413 [1.564, 3.262] a**
	Podospora	0.037 [0.000, 0.426]	0.057 [0.000, 0.430]	0.368 [0.087, 0.649]
	Polyscytalum	0.095 [0.04, 0.15]	0.074 [0.021, 0.127]	0.119 [0.08, 0.159]
	**Preussia**	**0.092 [0.000, 1.030] b**	**0.059 [0.000, 0.960] b**	**1.413 [0.735, 2.092] a**
	**Pseudogymnoascus**	**0.268 [0.07, 0.466] ab**	**0.549 [0.36, 0.739] a**	**0.314 [0.171, 0.457] ab**
	Pyrenochaeta	0.214 [0.072, 0.356]	0.114 [−0.022, 0.251]	0.104 [0.001, 0.207]
	Rhinocladiella	0.323 [0.221, 0.424]	0.320 [0.222, 0.417]	0.275 [0.201, 0.348]
	Schizothecium	0.135 [0.016, 0.254]	0.204 [0.09, 0.318]	0.275 [0.189, 0.361]
	Tetracladium	0.768 [0.510, 1.026]	0.741 [0.494, 0.989]	0.984 [0.798, 1.17]
	Thelebolus	0.033 [0.000, 0.544]	0.012 [0.000, 0.502]	0.578 [0.209, 0.947]
	Trichoderma	0.246 [0.156, 0.336]	0.197 [0.11, 0.283]	0.216 [0.15, 0.281]
	Verticillium	0.001 [0.000, 1.047]	0.000 [0.000, 0.000]	0.552 [0.000, 1.308]
	Xylaria	0.064 [0.000, 0.401]	0.323 [0.000, 0.647]	0.008 [0.000, 0.252]
Basidiomycota	Amanita	0.010 [0.000, 0.533]	0.500 [0.002, 1.002]	0.317 [0.000, 0.695]
	**Boletus**	**2.530 [0.787, 4.273] b**	**0.067 [0.000, 1.739] b**	**0.099 [0.000, 1.359] a**
	**Cortinarius**	**4.753 [0.000, 11.163] b**	**18.288 [12.136, 24.439] a**	**4.890 [0.257, 9.523] b**
	**Cryptococcus**	**2.637 [1.196, 4.078] a**	**0.545 [0.000, 1.927] b**	**1.142 [0.100, 2.183] ab**
	Entoloma	0.089 [0.000, 1.032]	0.837 [0.000, 1.742]	0.184 [0.000, 0.866]
	Geastrum	0.066 [0.000, 0.483]	0.059 [0.000, 0.460]	0.296 [0.000, 0.598]
	**Geminibasidium**	**2.174 [1.044, 3.304] a**	**0.346 [0.000, 1.43] b**	**0.966 [0.149, 1.782] ab**
	Hygrocybe	4.597 [1.402, 7.793]	0.326 [0.000, 3.393]	1.711 [0.000, 4.021]
	Inocybe	1.024 [0.195, 1.853]	0.319 [0.000, 1.115]	0.300 [0.000, 0.899]
	Mycena	0.141 [0.000, 0.322]	0.036 [0.000, 0.209]	0.124 [0.000, 0.255]
	Russula	0.946 [0.17, 1.721]	1.039 [0.295, 1.783]	0.866 [0.306, 1.426]
	**Scleroderma**	**0.734 [0.255, 1.213] a**	**0.464 [0.004, 0.923] ab**	**0.143 [0.000, 0.489] b**
	Sebacina	0.398 [0.000, 1.672]	1.151 [0.000, 2.373]	1.939 [1.019, 2.86]
	**Tomentella**	**0.769 [0.075, 1.462] ab**	**1.271 [0.605, 1.936] a**	**0.295 [0.000,0.796] b**
	Typhula	0.045 [0.000, 0.959]	0.744 [0.000, 1.621]	0.368 [0.000, 1.028]
	Xerocomellus	0.118 [0.000, 0.946]	0.793 [0.000, 1.588]	0.127 [0.000, 0.726]
Glomeromycota	Entrophospora	0.378 [0.000, 0.829]	0.199 [0.000, 0.631]	0.591 [0.266, 0.917]
Zygomycota	**Mortierella**	**3.172 [1.763, 4.58] b**	**3.793 [2.442, 5.145] b**	**6.174 [5.156, 7.192] a**

**Table A3:** Mean [−0.95 CI, 0.95 CI] relative abundance of functional guilds. Different lowercase letters and the bold font denote significant LSD difference α = 0.05 among low, mid, and high altitudes.

Functional Guilds	Low Altitude	Mid Altitude	High Altitude
**|** **Animal Parasite|-Animal Pathogen-Endophyte-Plant Saprotroph-Undefined Saprotroph**	**2.434 [1.716, 3.151] b**	**3.733 [3.044, 4.421] a**	**2.46 [1.941, 2.978] b**
**|Ectomycorrhizal|**	**8.418 [1.875, 14.96] b**	**22.94 [16.661, 29.219] a**	**6.818 [2.089, 11.547] b**
|Ectomycorrhizal|-Orchid Mycorrhizal-Root Associated Biotroph	0.398 [0.000, 1.672]	1.151 [0.000, 2.373]	1.939 [1.019, 2.86]
|Plant Pathogen|	1.704 [1.273, 2.134]	1.342 [0.929, 1.755]	1.806 [1.495, 2.117]
|Plant Saprotroph|-Undefined Saprotroph	1.045 [0.503, 1.587]	1.008 [0.487, 1.528]	1.538 [1.146, 1.929]
|Undefined Saprotroph|	2.479 [1.284, 3.674]	0.882 [−0.265, 2.029]	1.69 [0.826, 2.553]
|Undefined Saprotroph|-Undefined Symbiotroph	4.609 [1.412, 7.807]	0.345 [−2.723, 3.414]	1.848 [−0.463, 4.159]
**Animal Pathogen-Dung Saprotroph-Endophyte-Epiphyte-Plant Saprotroph-Wood Saprotroph**	**1.784 [1.055, 2.513] ab**	**1.097 [0.397, 1.797] b**	**2.157 [1.63, 2.684] a**
**Animal Pathogen-Endophyte-Fungal Parasite-|Undefined Saprotroph|**	**7.237 [5.899, 8.574] a**	**6.652 [5.368, 7.936] a**	**4.369 [3.402, 5.336] b**
Bryophyte Parasite-Dung Saprotroph-Ectomycorrhizal-Fungal Parasite-Leaf Saprotroph-Plant Parasite-Undefined Saprotroph-Wood Saprotroph	0.596 [0.000, 1.774]	0.99 [0.000, 2.120]	1.299 [0.448, 2.151]
**Dung Saprotroph-Endophyte-Lichen Parasite-|Plant Pathogen|-Plant Saprotroph-Undefined Saprotroph-Wood Saprotroph**	**0.897 [0.000, 2.071] b**	**0.557 [0.000, 1.684] b**	**2.413 [1.564, 3.262] a**
Endophyte	0.735 [0.424, 1.047]	1.13 [0.832, 1.429]	1.074 [0.849, 1.299]
**Endophyte-|Plant Pathogen|-Plant Saprotroph-Undefined Saprotroph**	**1.12 [0.104, 2.137] b**	**1.198 [0.223, 2.174] b**	**3.66 [2.925, 4.395] a**
**Endophyte-Plant Saprotroph-|Undefined Saprotroph|**	**3.172 [1.764, 4.579] b**	**3.804 [2.453, 5.155] b**	**6.174 [5.156, 7.191] a**
**Plant Pathogen-Undefined Saprotroph-|Wood Saprotroph|**	**2.046 [1.165, 2.927] b**	**3.711 [2.865, 4.557] a**	**2.309 [1.672, 2.946] b**
Undefined Saprotroph	1.254 [0.000, 3.724]	1.369 [0.000, 3.739]	3.044 [1.259, 4.829]

**Table A4:** Distance-based redundancy analysis of common and rare fungal communities against environmental variables. SS, the sum of squares; TN, soil total nitrogen; TP, soil total phosphorus; EC, electrical conductivity. Values in bold are significant.

Variables	Common Taxa		Rare Taxa		All Taxa	
	SS	*p*	SS	*p*	SS	*p*
Altitude (m)	**2.242**	**0.001**	**1.573**	**0.001**	**2.179**	**0.001**
Soil temperature (°C)	0.731	0.064	**1.077**	**0.008**	0.776	0.053
Soil moisture (v v^−1^)	**1.139**	**0.001**	**1.118**	**0.004**	**1.164**	**0.001**
pH	**1.943**	**0.001**	**1.192**	**0.001**	**1.869**	**0.001**
TN (g kg^−1^)	**1.023**	**0.002**	**1.136**	**0.003**	**1.023**	**0.001**
TP (g kg^−1^)	**0.846**	**0.012**	**1.055**	**0.029**	**0.862**	**0.019**
C:N ratio	0.536	0.491	1.000	0.155	0.570	0.550
Bulk density (g cm^−3^)	0.680	0.094	0.975	0.415	0.699	0.152
Clay content (%)	**0.817**	**0.020**	1.024	0.126	**0.865**	**0.017**
Silt content (%)	0.648	0.185	**1.131**	**0.001**	0.695	0.135
EC (ds cm^−1^)	**0.897**	**0.008**	0.952	0.705	**0.892**	**0.017**
Herbaceous richness	**1.215**	**0.001**	**1.208**	**0.001**	**1.232**	**0.001**
Residual	58.422		102.582		63.397	

**Table A5:** Fungal co-occurrence networks at low, middle, and high altitudes.

Topologies	Low Altitude	Mid Altitude	High Altitude
Number of species	986	972	808
Positive edges	3096	2159	1476
Negative edges	4	15	9
Negative/positive edges (%)	0.13	0.69	0.61
Average degree	6.28	4.47	3.68
Diameter	17	15	20
Clustering coefficient	0.38	0.32	0.29
Number of modules	38	42	58
Modularity	0.67	0.73	0.77
